# Fang Filling

**Published:** 1859-11

**Authors:** A. Berry


### FANG FILLING.

It is much easier to *talk* of filling to the apex of a fang than to *do* it. No clairvoyant can inform the operator when the gold reaches the extremity of the nerve canal so as to fill it completely. I have sometimes filled the fangs of teeth, but doubt whether I have ever succeeded in filling them entirely to their apices. Others may succeed better; but probably nearly all the teeth so filled when extracted, as unfortunately they sometimes must be, show that a portion of their nerve cavities was not filled.

Several years ago, while conversing with Dr. Samuel Griffith, of Louisville, on treating teeth that had lost their vitality, or had their nerves destroyed, by removing the membranes, &c., in the pulp cavity, he described a method of drilling a small opening under the edge of the gum to the cavity, in which opening a piece of hickory is to be inserted, and the lower portion of the canal and the cavity in the crown filled, when the hickory is to be removed to allow the escape of any matter accumulating in the nerve canal. A patient in the Dr's chair, inserting a pin in an opening under the gum in the fang of a central incisor, said, "Here is a tooth filled in that way by Dr. ——, of Philadelphia, five years ago, which has given me no trouble since."

My observation leads me to believe that this mode of treating such cases is more successful than fang filling in preventing alveolar abscess.

A. BERRY.

